# Miscellanies

**Published:** 1836-10-01

**Authors:** 


### MISCELLANIES.

### An Act to provide for the Attendance and Remuneration of Medical Witnesses at Coroners' Inquests.

[Aug. 18th, 1836.]

*Summoning of Medical Witnesses.*

1. Whereas it is expedient to provide for the attendance of Medical Witnesses at Coroners' Inquests, also Remuneration for such attendance, and for the performance of Post-mortem Examinations at such Inquests: Be it therefore enacted by the King's most Excellent Majesty, by and with the advice and consent of the Lords Spiritual and Temporal, and Commons, in this present Parliament assembled, and by the authority of the same, that from and after the passing of this Act, whenever upon the summoning or holding of any coroner's inquest it shall appear to the coroner that the deceased person was attended at his death, or during his last illness, by any legally qualified medical practitioner, it shall be lawful for the coroner to issue his order, in the form marked (A) in the Schedule hereunto annexed, for the attendance of such practitioner as a witness at such inquest; and if it shall appear to the coroner that the deceased person was not attended at or immediately before his death by any legally qualified medical practitioner, it shall be lawful for the coroner to issue such order for the attendance of any legally qualified medical practitioner, being at the time in actual practice in or near the place where the death has happened; and it shall be lawful for the coroner, either in his order for the attendance of the medical witness, or at any time between the issuing of such order and the termination of the inquest, to direct the performance of a post-morten examination, with or without an analysis of the contents of the stomach or intestines, by the medical witness or witnesses who may be summoned to attend at any inquest; provided that if any person shall state upon oath before the coroner that in his or her belief the death of the deceased individual was caused partly or entirely by the improper or negligent treatment of any medical practitioner or other person, such medical practitioner or other person shall not be allowed to perform or assist at the post-mortem examination of the deceased.

*Additional Medical Evidence if the first be not satisfactory.*

II. And be it further enacted, That whenever it shall appear to the greater number of the jurymen sitting at any coroner's inquest, that the cause of death has not been satisfactorily explained by the evidence of the medical practitioner or other witness or witnesses who may be examined in the first instance, such greater number of the jurymen are hereby authorized and empowered to name to the coroner, in writing, any other legally qualified medical practitioner or practitioners, and to require the coroner to issue his order, in the form herein-before mentioned, for the attendance of such last-mentioned medical practitioner or practitioners as a witness or witnesses, and for the performance of a post mortem examination, with or without an analysis of the contents of the stomach or intestines, whether such an examination has been performed before or not; and if the coroner, having been thereunto required, shall refuse to issue such order, he shall be deemed guilty of a misdemeanor, and shall be punishable in like manner as if the same were a misdemeanor at common law.

*Fees to Medical Witnesses and Funds for their Payment.*

III. And be it further enacted, That when any legally qualified medical practitioner has attended upon any coroner's inquest in obedience to any such order as aforesaid of the coroner, the said practitioner shall for such attendance at any inquest in Great Britain be entitled to receive such remuneration or fee as is mentioned in the table marked (B.) in the Schedule hereunto annexed; and for any inquest held in Ireland, the said practitioner shall be paid in the manner provided by the laws in force in that part of the United Kingdom; and the coroner is hereby required and commanded to make, according to the form marked (C.) in the Schedule hereunto annexed, his order for the payment of such remuneration or fee, when the inquest shall be held in Great Britain, and such order may be addressed and directed to the churchwardens and overseers of the parish or place in which the death has happened; and such churchwardens and overseers, or any one of them, is and are hereby required and commanded to pay the sum of money mentioned in such order of the coroner to the medical witness therein mentioned, out of the funds collected for the relief of the poor of the said place.

*Fee not payable for a post-mortem Examination instituted without Order from the Coroner.*

IV. Provided nevertheless, and be it further enacted, That no order of payment shall be given, or fee or remuneration paid, to any medical practitioner for the performance of any post-mortem examination which may be instituted without the previous direction of the coroner.

*Inquests on Bodies of Persons dying in public Institutions.*

V. Provided also, and be it further enacted, That when any inquest shall be holden on the body of any person who has died in any public hospital or infirmary, or in any building or place belonging thereto, or used for the reception of the patients thereof, or who has died in any county or other lunatic asylum, or in any public infirmary or other public medical institution, whether the same be supported by endowments or by voluntary subscriptions, then and in such case nothing herein contained shall be construed to entitle the medical officer whose duty it may have been to attend the deceased person as a medical officer of such institution as aforesaid to the fees or remuneration herein provided.

*Penalty for neglecting to attend.*

VI. And be it further enacted, That where any order for the attendance of any medical practitioner as aforesaid shall have been personally served upon such practitioner, or where any such order not personally served shall have been received by any medical practitioner in sufficient time for him to have obeyed such order, or where any such order has been served at the residence of any medical practitioner; and in every case where any medical practitioner has not obeyed such order, he shall for such neglect or disobedience forfeit the sum of five pounds sterling, upon complaint thereof made by the coroner or any two of the jury before any two justices having jurisdiction in the parish or place where the inquest under which the order issued was held, or in the parish where such medical practitioner resides; and such two justices are hereby required, upon such complaint, to proceed to the hearing and adjudication of such complaint, and if such medical practitioner shall not show to the said justices a good and sufficient cause for not having obeyed such order, to enforce the said penalty by distress and sale of the offender's goods, as they are empowered to proceed by an act of parliament for any other penalty or forfeiture.

*Act not to extend to Scotland.*

VII. And be it enacted, That nothing in this Act contained shall extend to Scotland.

---

SCHEDULE TO WHICH THIS ACT REFERS.

(A.) *Form of Summons.*

Coroner's inquest at , upon the body of

By virtue of this, my order, as coroner for , you are required to appear before me and the Jury at , on the day of 18 , at o'Clock, to give evidence touching the cause of death of , [*and then add, when the witness is required to make or assist at a post-mortem examination*] and make or assist in making a post-mortem examination of the body, with [*or* without] an analysis, [*as the case may be,*] and report thereon at the said inquest.

(Signed) Coroner.

To , Surgeon, [*or* M.D., *as the case may be.*]

(B.) *Table of Fees.*

1. To every legally qualified medical practitioner for attending to give evidence under the provisions of this Act, at any coroner's inquest, whereat no post-mortem examination has been made by such practitioner, the fee or remuneration shall be one guinea.
2. For the making of a post-mortem examination of the body of the deceased, either with or without an analysis of the contents of the stomach or intestines, and for attending to give evidence thereon, the fee or remuneration shall be two guineas.

(C.) *Coroner's Order for the payment of Medical Witnesses.*

By virtue of an act of parliament passed in session of holden in the intituled I, the coroner of or for do order you, the overseers of the parish [*or* township, *as the case may be*], to pay to the sum of [one guinea *or* two guineas, *as the case may be*], being the fee [*or* fees] due to him for having attended as a medical witness at an inquest holden before me this day of , upon the body of , about the age of , who was found dead at , [*or other particulars or description*], and at which said inquest the jury returned a verdict of

(Signed) Coroner.

Witness by me of

To the Overseers, &c. &c."

So few are the boons accorded to the medical profession by the Legislature, that even this one, small as it is, deserves gratitude—and thanks are due to the honorable Member for Finsbury for his assistance in procuring it. Let it be well remembered that, now when the service of medical men is compulsory and paid for, it behoves each and every member to take care that he be qualified to give evidence in a court of law. He cannot now shrink from the duty by sending a substitute to stand the brunt of a cross-examination. We would advise old and young to study medical jurisprudence with more care than they have hitherto done. Sat verbum.

---

PROVINCIAL MEDICAL AND SURGICAL ASSOCIATION.

The members of the *Bristol* Council have taken into consideration the proposed connexion between the Eastern Society and the Provincial Medical and Surgical Association, and are of opinion that it is at direct variance with the great principle of the Association and destructive of that unity of design and operation so essential to its efficiency; and moreover that it would tend to embarrass without proportionally benefitting the general arrangements of the Association.

The Council are not aware of any peculiar advantages attending a partial connexion that would not be better attained by the complete junction. It has been urged indeed that the Association is becoming "too numerous" and the duties consequently devolving, too heavy to be discharged by our honorary and excellent secretaries. The Council cannot imagine that numbers can be regarded as an evil—every addition of a name to the society they have been accustomed to hail as an accession of strength and respectability and indicative of the increasing prosperity of the Association.

But admitting the validity of the objection, it is quite obvious that the proposed connexion would complicate rather than simplify the affairs of the Association, rendering necessary separate books, accounts, &c. Whereas, on the other hand, were the Association so fortunate as to embrace the whole of the provinces (by no means a problematical supposition), it is presumed that our secretaries under the present constitution, would be found equal to such augmented duties.

Considerations of economy and still more a regard to the strict maintenance of that harmony which has hitherto characterised all the proceedings of the Society are strongly in favor of the latter arrangement. It may not have occurred even to the members of the Council, that by the terms actually suggested (two thirds of the subscriptions of the Eastern Society being paid into the general treasury) the remaining one-third, that is, one hundred in every three hundred pounds, may be considered as virtually lost to the Association, and moreover that confusion would be liable to arise from the introduction of two classes of members with different rates of subscription.

Under the influence of these impressions, the Council are decidedly in favor of an absolute union: and are convinced that, unless such junction be effected, the general interests of the Association will be better promoted by its remaining as heretofore a distinct and independant Society. At the same time the Council are anxious to obtain the co-operation of so large and influential a body of practitioners; should therefore our Eastern brethren upon reconsideration be disposed to adopt the more comprehensive view of the question; and in compliance with the sentiments so generally entertained and expressed by the several local Councils, join our ranks as common members of one Association, we shall be most happy to hold out the right hand of fellowship.

Signed on behalf of the Council,
WILLIAM HETLING.

*Bristol*, 13*th July*, 1836.

*Remarks*. Matters having come this length, we see little chance of a union of the two Associations. The younger branch having tasted independence, and enjoyed a separate existence, will hardly give these up, and suffer itself to be merged in the parent institution. We would suggest that the two Associations, if they remain distinct, should meet every fourth year in the metropolis, to join their brethren of the capital in scientific communication, and friendly intercourse. It is a pity, indeed, that any distinction should be made between town and country, in medical associations. We are inclined to agree with our contemporary of the Lancet, that a National Association of Medical Practitioners, meeting sometimes in London, and at others, in the large provincial towns, would be preferable to the present plan.

---

### NEW FARTHING CLUB; OR METROPOLITAN INFINITESIMAL DISPENSARY.

A prospectus of this important institution has just reached us. It is to be under the direct patronage of a Prince of the Blood, a distinguished EARL, and many of the nobility and gentry. Its own merits, however, will insure it universal approbation and support. The superintendence of the Dispensary is placed in the hands of four of the most experienced HOMŒOPATHISTS of this country. It is on a plan so much superior to the penny-clubs, now spreading over the country, that it will soon supersede them all. The subscription is to be only *one farthing* per head per annum! We may calculate that, taking the rich with the poor, there is an annual expenditure, in London, of twenty shillings per head, on medical attendance and medicines—and as the Metropolitan Dispensary will do away entirely with the heavy fees and long bills of physicians, surgeons (excepting for operations), apothecaries, and chemists, the saving to the public of this capital will be more than a MILLION ANNUALLY!!

There can be no doubt that the whole population will subscribe at once; but, under such patronage, a charter of incorporation will certainly be obtained next session, and thus its success will be secure.

Four homœopathic laboratories are to be established, namely, at the Chelsea Water Works, on Primrose Hill—at the New River Head—at South Lambeth—and at Hampstead. From these sources infinitesimal doses of the choicest homœopathic remedies will be daily issued, not only for the cure, but for the prevention of all diseases. Thus it is calculated that, when scarlatina breaks out, a daily solution of twenty grains of belladonna in the Chelsea reservoir, will protect all individuals (within the range of that company) against the scarlet fever, besides curing those who have actually caught the disease. If cholera re-appears, a lump of camphor is to be thrown into each of the four reservoirs, the emanations from which will soon check the Asiatic pestilence.*

The great beauty of the infinitesimal system is this, that each remedy will only grapple with, or prevent those diseases that are of its own nature—"SIMILIA SIMILIBUS CURANTUR." Thus a few grains of aconite daily dissolved in each of the reservoirs, will cure or prevent all the aconite diseases of this overgrown metropolis, without producing any effect on those in health, or modifying any other diseases or remedies.

* See Dr. Uwins' Pamphlet.

The saving of money will be the smallest part of the benefits which must flow from this most fortunate discovery. In a few years, all diseases, excepting those resulting from casualties, will disappear, or nearly so, and the value of life will be so enhanced, that the insurance offices will be able to reduce their premiums nearly one half! The grief, anxiety, and sorrow of families, for the loss of children, &c. will be prevented, as all people will then die the death of Nature, far beyond the obsolete boundary of three-score years and ten.

It is true, however, that there is no unmixed good in this world. Certain orders of society, now flourishing, will be reduced to beggary. The Apothecaries' Company (who are getting up a petition to Parliament against homœopathy) will be ruined by the "atomic doses" of the Infinitesimal Dispensary. The College of Physicians, being ruined already, think it useless to remonstrate. The College of Surgeons, though they will still have the monopoly of operations, are preparing, nevertheless, to petition the legislature, because they well know that, though surgery is their *profession*, physic is the best part of their *practice*. The apothecaries may, of course, shut up shop—and all those beautiful drug-palaces, whose windows, at night, look like constellations of etherial fire—

"flammisque imitante pyropo"—

must be converted into gin-shops or soda-water manufactories. It is to be hoped that Government may, in charity, grant a free passage to Australia for, at least, twenty thousand ALLOPATHICS, who will thus be thrown out of work in this metropolis.

These are little specks of evil attendant on a public—a universal blessing. The homœopathic superintendants have humanely determined to station one of their elèves, with a few small vials and pots, some miles beyond Oxford, in order to medicate the stream of the Thames, for the benefit of the University, Maidenhead, Windsor, Kingston, Richmond, and the various towns along the river as far as Gravesend. It is calculated that a scruple of oxymuriate of mercury and three pints of fluid extract of sarsaparilla thrown into the Thames at Fulham, will prove a powerful alterative for all those who drink the water supplied by the Chelsea and South Lambeth Companies. A much smaller quantity will do for the New River Head. Infinitesimal Dispensaries will, without doubt, be established in all the great cities and towns of England. And, as the present race of practitioners will certainly be the last of their kind, so our great medical schools, and even our hospitals will shortly vanish from the scene. Homœopathic doses of medicine will naturally require but "atomic doses" of the science on which medicine depends. Indeed we see no reason why, on the homœopathic system, we should undergo the drudgery of studying anatomy, physiology, therapœia, or chemistry. A very few medicaments, divided and subdivided into inappreciable tenuity, are endued with the power of penetrating the most minute tissues of the human fabric—with the instinctive sagacity—the more than chemical affinity, of searching out, seizing, and coalescing with their "SIMILITUDES" in these remote recesses,—and there neutralizing their morbid qualities, and expelling them ultimately from the body! If this be not the millennium of medicine, we can form no idea of what a millennium is! HOMŒOPATHY has, we conceive, brought us to the verge of this happy epoch—and the infinitesimal physician is entitled to the infinite gratitude of mankind!

P.S. We have just learnt a piece of curious information. It is this:—ever since the new Poor Law Act came into operation, a celebrated homœopathist, of this metropolis, has been giving lectures on the infinitesimal art, to pupils who have "passed the Hall," and who are qualifying for the office of "contract doctors" throughout the provincial districts. We have now a solution of the low rates at which these UNDERTAKERS contract to attend the sick poor. The homœopathic system will enable these practitioners to beat the ALLOPATHICS clean out of the field! The training requires about six weeks, and the whole apparatus medicaminum consists of a few bottles of medicated mustard seed, labelled "homœopathic pills." Our informant shewed us a single formula, and the *mode* of preparation is the same in all. Five grains of extract of aconite are dissolved in a pint of distilled water, and the solution is poured off clear. Into this is put a pint of white mustard seed. In a few hours the seeds swell and absorb the whole of the solution. They are then spread out in the open air, when they soon shrink back to their original dimensions, being, however, impregnated with the aconite, and fit for use. The dose is from 1 to 10 of these aconite pills. The mustard seed is medicated or imbued with all other articles of the homœopathic pharmacopœia in the same way. The cost of physicing a large parish on the "atomic plan," will, it is supposed, not exceed half-a-crown per annum.

---

### HÆMORRHAGE FROM THE MEDULLA OF THE HUMERUS AFTER AMPUTATION.*

Mr. Badderly communicates this brief notice of the case to our contemporary.

On the 23rd December, 1835, a gunner of the horse artillery, in the act of springing his staff, had his fore-arm so shattered by the sudden explosion of the gun, that it became necessary to amputate it above the elbow joint, which was performed 20 minutes after the accident.

A troublesome hæmorrhage supervened; and, on opening the stump two hours after, to discover the source, it was found to originate from the medulla of the bone, from which joint was seen an arterial stream of the size of a small crowquill, which resisted a variety of methods adopted to check it, nor was it till after the marrow had been destroyed to the depth of a quarter of an inch by the actual cautery, that the bleeding entirely ceased.

At the termination of a week, there had been no recurrence of the hæmorrhage, and the stump was suppurating.

---

* India Journ. March 1, 1836.

### Sixteenth Report of the Dundee Lunatic Asylum.

(Effect of Fright.)

The following short extract from this report embraces a useful caution as respects the treatment of young people, whether at school, or during their apprenticeship.

We admitted an interesting little boy, aged fifteen years ;—when twelve years of age he was placed as an apprentice to a light, clean business. A trifling article being one day amissing, his master locked up all his apprentices in a dark coal-cellar. The children became dreadfully afraid. They were let out soon, with the exception of this unfortunate patient, who was detained till past midnight. From this time he became extremely nervous, melancholy, and for some time past has been imbecile. He will be for life a burden to his relatives and friends. It may be here mentioned, that the lost utensil was found in a safe place early on the following morning, which completely exculpated the boys. His forehead was extremely deficient, unlike the rest of his family.

The report itself is satisfactory, and creditable to the professional talents of Dr. Nimmo and Mr. Macintosh.

---

### Medical Clubs.

We have often adverted to the redundancy of the medical profession, and the misery as well as mischief which are likely to result from such plethoric state of our ranks. The proposals of club societies or dispensaries, are one of the signs of the times, and closely connected with the redundancy in question. There are now so many hands out of work, that HEADS will be exerted in devising some new means of providing provender for the belly. The consequence will be, and, we fear, must be, that there will always be found, in the medical profession, a sufficient number of individuals who will embrace any plan, however ignoble, that may promise the means of even a wretched subsistence. The contract doctors, under the new poor-law system, offer an illustration—and the penny-clubs another. That many who propose these dispensaries have good intentions, we have no doubt. No one, for instance, can suppose that such a man as Dr. Hodgkin, would approve of them, if he did not, in conscience do so. But men's intentions may be good, and the result of their actions may be evil. This will be the case, in the plan of Mr. Smith and others for self-supporting dispensaries. Even if these institutions could be kept to their original boundaries, much detriment will accrue to the medical practitioner, whose avocations may lie among the humbler classes of society. But the circle will daily spread, till it embraces a very considerable portion of the middling classes, who now furnish a fair living for the general practitioner.

Mr. Smith proposes that a dispensary should be established in each market town and large village, for the supply of medicines and attendance to those "who cannot afford to pay *in the usual manner*, for advice and drugs." What kind of indefinite line is this? Again, those who are to be admitted on the free class, and who pay annual subscriptions, are to be so admitted "on obtaining a proper recommendation." Every body knows how easily a recommendation may be obtained. Great numbers, who are well able to pay the doctor, will get on this free-class list, at the expense of a mere trifle—and still greater numbers will obtain entirely gratuitous assistance, who are now able to contribute something to the junior medical practitioner, who is obliged to work his way up from one class to another. Thus one medical man, for a yearly income of, perhaps, not more than one hundred pounds, will take the bread out of the mouths of three or four other men, who might have received two or three hundred pounds each per annum from the same circle!!

This, in itself, would be a serious evil, but it would not constitute a hundredth part of the injury which the profession would sustain from this system. No sooner will these clubs be established for the labourer and pauper, than will others rise among the artizans, shop-keepers, and tradesmen. We have, at this moment a plausible pamphlet lying on our table, projecting clubs for all classes, from the peer to the peasant, on very easy terms. It is calculated that, in the army and navy, when soldiers and sailors are not exposed to more than the common sources of sickness, there will be usually about 5 per cent. on the sick-list. Estimating the proportion at double this—or even 15 per cent., in private life—and supposing that one hundred families club together for medicines and medical assistance, at the rate of twenty shillings per head—and supposing that the families average six individuals each, here will be an annual income of £600 for a medical man! The expense of medicines, in all probability, will not exceed one hundred pounds, at the very utmost, so that there will be a clear income of 500 pounds per annum! How many hundreds of our starving brethren would jump at such a scheme! This, we prognosticate, will be the result of the penny-clubs, as they are called. They will prove the root of evil, from which branches will shoot aloft, and adumbrate the whole field of medicine. All admonitions—all appeals to a sense of honour—or a sense of respect for the profession, will be vain. There will always be such an overplus in the ranks of medical society, that numerous volunteers will be ever ready to enter for this degrading service. There is but one remedy—one preventive check—in our humble opinion; but it will be condemned by our ultra-liberal brethren, who advocate the removal of every impediment to the ingress of all aspirants for medical fame and fortune. We have, however, often broached the subject before. It is the extension of medical education to its utmost limits. There is now a redundancy; and why?—Because it is calculated by parents and guardians that the entrance into the medical profession is cheaper and more certain than into the church, the law, or other genteel walks of life. This impression must be removed by shewing them that the expense of time and money will be much greater than it has hitherto been,—otherwise the influx will continue to increase—the club-system will spread, and two-thirds of the rising generation of practitioners will be swamped!

This club-system, in fact, is rapidly extending through all grades of society, and is one mark of a redundant population. We see a thousand people combine together, to erect a fine house, and have dinners and wine at a very cheap rate. It is well ascertained that not a fourth of the subscribers will ever, *at any one time,* make use of the club—and the subscriptions of the other three-fourths keep up the establishment, and enable the actual club-goers to live in luxury and at little expense. Will this system not extend to physic as well as to food? That it will! The Hebrews, who are a knowing and economical race, have long acted on this system. A great proportion of the Israelites belong to clubs, where a medical man forms one of the contracting parties. The march of intellect and the *res angusta domi* of a redundant population, will convert some millions of Christians, in this country, into Jews, as far as medical clubs and contracts are concerned. It is not generally known, but we know it, for a certainty, that several physicians in this metropolis, who hold their heads high in the profession, are in the habit of attending families by the year, for a moderate sum. What is this but a CLUB on a small scale?—Those who embark in this destructive club-system, may subsist for a while, at the expense of their neighbours; but ultimately the ruin will reach themselves, and the attempt to retrace their steps will be vain.

We have said nothing of its detrimental effects on the *science* of medicine. It will be as injurious to that as to the respectability of its professors. This struggle to physic people cheap, will take away the stimulus to discovery. The contract being *fixed,* the great object is to discover the *cheapest* way to work, and expensive medicines will seldom be employed. We remember a navy surgeon of the old regime, who had one bottle of medicine for the starboard, and another for the larboard watch. The first question asked, when a man came down to cockpit was—"What watch do you belong to?"—"Starboard, Sir." "Give him a dose out of No. 1." These bottles contained little else than sea-water and Epsom salts. One day, the doctor fell overboard. An arch quarter-master ran up to the officer on watch, and told him the doctor had fallen into his own medicine-chest. The contract doctors will probably have a bottle for each parish of the union—or they may even go so far as to have a differently-coloured bottle for the females. There will be great advantage in nauseous medicine, in these cases. The paupers will have as great a dread of it as of the work-house—perhaps greater.

---

### PATRONAGE OF QUACKS.

We thought that, in England, quackery flourished with unexampled vigour.—But we were mistaken. It is rarely that the quack here is able to entrap the regular physician or surgeon into an open patron, certifying under his hand and seal the mighty virtues of an unknown composition, vended for gain, and for gain alone. Not so beyond the Atlantic! We were utterly astonished to find an impudent PANACEA bolstered up with the names and certificates of some of the first authorities, in the medical profession, of the United States! We were thunderstruck on perusing such documents as the following—some twenty or thirty being obtained by a noted nostrum-vender in the land of freedom.

"CERTIFICATES OF RECOMMENDATION.

FROM DOCTOR N. CHAPMAN,
*Professor of the Institutes and Practice of Physic and Clinical Practice in the University of Pennsylvania, President of the Academy of Medicine of Philadelphia, &c.*

I have within the last two years had an opportunity of seeing several cases of very inveterate ulcers, which, having resisted previously the regular modes of treatment, were healed by the use of Swaim's PANACEA; and I do believe, from what I have seen, that it will prove an important remedy in scrofulous, venereal, and mercurial diseases.

N. CHAPMAN, M.D.

FROM DOCTOR W. GIBSON,
*Professor of Surgery in the University of Pennsylvania, Surgeon and Clinical Lecturer to the Alms-House Infirmary, &c. &c.*

I have employed the PANACEA of Mr. Swaim, in numerous instances, within the last three years, and have always found it extremely efficacious, especially in secondary syphilis and in mercurial disease. I have no hesitation in pronouncing it a medicine of inestimable value.

W. GIBSON, M.D.

FROM DOCTOR VALENTINE MOTT,
*Professor of Surgery in the University of New York, Surgeon of the New York Hospital, &c. &c.*

I have repeatedly used Swaim's PANACEA, both in the Hospital and in private practice, and have always found it to be a valuable medicine in chronic, syphilitic, and scrofulous complaints, and in obstinate cutaneous affections.

VALENTINE MOTT, M.D.

FROM DOCTOR WILLIAM P. DEWEES,
*Adjunct Professor of Midwifery in the University of Pennsylvania, &c.*

I have much pleasure in saying I have witnessed the most decided and happy effects in several instances of inveterate disease from Mr. Swaim's PANACEA, where other remedies had failed—one was that of Mrs. Brown.

WM. P. DEWEES, M.D."

Eheu, jam satis! We are mortified and grieved, beyond measure, to find professional *propriety* (to give it no other name) at so low an ebb among our brethren in America! This admonition from Europe will surely rouse the faculty of the United States to some sense of the duty they owe to their brethren throughout the world.

---

### Spontaneous Combustion.

An instance of spontaneous combustion is reported in the French papers, to have taken place at Aunay, in the department of Avalon. A very fat woman, aged 74 years, and addicted to drinking brandy at 27 degrees, lived alone, and one evening returned home as usual, but, as she did not appear among her neighbours the next morning, they knocked at her door. No answer being returned to repeated demands, they summoned the mayor, who forced the door, and exposed a horrible spectacle, accompanied by an extraordinary smell. Near the chimney laid a heap of something burnt to cinders, at one end of which was a head, a neck, the upper part of a body, and one arm. At the other end were some of the lower parts, and one leg, still retaining a very clean shoe and stocking. No other traces of fire were to be seen, except a blue flame which played along the surface of a long train of grease, or serous liquor, which had been produced by the combustion of the body. The mayor found it impossible to extinguish this flame, and summoned all the authorities; and, from the state of the apartment and comparison of circumstances, it was concluded among them that, previous to going to bed, for which she had evidently been making preparations, the woman had been trying to ignite some embers with her breath. The fire communicating with the body by means of the breath, combustion probably took place, and would appear to confirm an opinion entertained by several learned men, that that which is called spontaneous combustion of the human frame, never takes place without the presence of some ignited body near the person predisposed to combustion. A surgeon who bled an habitually drunken person, accidentally put the blood extracted near a candle, when immediately a blue flame appeared on the surface, which he found extremely difficult to extinguish.*

There is nothing in the preceding case inconsistent with probability, whatever might have been the actual fact. Numerous experiments have shewn that the expired pulmonary vapour becomes strongly charged with volatile substances taken into the system, and alcohol, phosphorus, &c. are readily detected in it. The foul breath of persons addicted to improper feeding is not merely the consequence of depraved secretions in the air-passages, but of the exhalation of odorous matters from pulmonary vessels. There have been now so many instances of spontaneous combustion placed on record, that it is impossible to doubt their occurrence. But they almost all happen on the Continent, and the majority, if our memory serves us, in France.

---

### Tunnels on Rail-Roads.

A question of some importance, as respects the health of passengers travelling on rail-roads, was lately discussed before a Committee of the House of Lords. Several medical gentlemen were examined and cross-examined, as to the probable effects of passing rapidly through long tunnels, placed at a considerable distance below the surface of the earth. The inconveniences or dangers of these subterraneous transits may be contemplated under three different points of view: viz. the transitions of temperature —impurity of air—and the reverberations of sound, as affecting the nervous system of sensitive travellers or invalids. It is well known that, at a depth of 80 feet below the surface of the earth, the temperature remains stationary through Summer and Winter, and is about the mean temperature of the climate. In this country, therefore, the temperature of the Brighton tunnels would be about 52° or 53°. In Summer, then,

---

* Athenæum, July 30th.

when the thermometer stood at 76°, and in Winter, when it stood at 32°, the transition would be 20° at the entrance, and 20° back again at the exits. The question is, will a sudden vicissitude of 20 thermometrical degrees be safe? We rarely experience such a vicissitude in this climate, even when spread over a whole day, and yet we are accustomed to consider atmospheric transitions as a source of much illness in England. But, it may be said, this is not worse than going out of a crowded assembly into the cold night air. This may be true; but it does not lessen the objection, because there is hardly a more fertile source of pulmonic, rheumatic, and neuralgic affections than these very occurrences. Besides, it is questionable whether there is ever a transition of 20° in going out or in of crowded theatres or assembly-rooms.

In mitigation of the objections to the tunnels, it has been urged that, on the Brighton road, the transit will occupy only about a minute, and that, during that time, the inside passengers could keep the windows closed. The transit would certainly be less hazardous to the inside passengers than to those on the outside. We imagine, however, that the heat of the engine and the fumes from the coke will raise the temperature at least ten degrees, besides proving very suffocating in a narrow and confined space. Thus, in Winter, the passengers might plunge from 32° to 62°—and, on emerging, would plunge at once from an atmosphere of 62° to one of 32°—or 30 degrees' difference of the thermometer.

How far the air of a tunnel, half a mile in length, may become vitiated by the breathing of passengers and the emanations from coke, we cannot pretend to say. Neither can we say whether or not a free ventilation could be secured in such a long trajet. Sir A. Carlisle and Dr. A. T. Thomson were of opinion, that the air would become more and more vitiated, and that it would be nearly stagnant. Dr. J. Johnson declined to give an opinion on this point. As the train must displace a volume of air equal to itself at each transit, besides producing a certain degree of current, it is probable that the stagnation and vitiation of the air may not be so great as is imagined.

The effects of reverberation of sound on sensitive people, while passing the tunnels at a rate of 25 miles an hour, were alluded to only by Dr. Johnson, who quoted a passage bearing on this point, from his own description of the Manchester Rail-road, published two years previously. He was cross-questioned by Counsel on this point, by referring to the Grotto of Posillipo, near Naples; but the comparison was very far from being *ad rem*.

The cross-examining junior Counsel then ingeniously shifted his ground to the atmospheric vicissitudes, and quoted a passage from one of Dr. Johnson's own tours among the Alps, where a transition of more than 50 degrees was experienced between Martigny and the Great St. Bernard, without any ill effects. The fact was acknowledged, and no comment was made at that time by the witness. The opposing Counsel afterwards took care to draw forth an explanation. The Alpine transition was spread over a space of 28 miles of laborious ascent, and occupying a period of nearly six hours. Besides, the tourists were accustomed to great transitions, and were fortified against them by daily exercise in the open air. Such a transition, occurring between London and Brighton, would be very apt to prove highly detrimental to tender invalids, or people affected with pulmonary or rheumatic complaints.

Considering, however, how much doctors differ on all points of theory or practice, it is rather surprising that a cloud of medical witnesses were not brought before their Lordships, to prove that atmospheric vicissitudes are innocuous, and the trajet of tunnels are beneficial to health, as affording shelter from sun, rain, and cold.

